# Efficacy and safety of blood-activating herbs combined with edaravone in the treatment of acute ischemic stroke: A protocol for systematic review and meta-analysis

**DOI:** 10.1097/MD.0000000000032162

**Published:** 2022-12-02

**Authors:** Zhuoyi Su, Shuai Zhang, Ziqiao Yu, Hong Jin, Weichen Sun, Ziqi Yang, Dexi Zhao

**Affiliations:** a Department of Traditional Chinese Medicine, Changchun University of Chinese Medicine, Changchun, China; b Jilin Ginseng Academy, Changchun University of Chinese Medicine, Changchun, China; c Department of Acupuncture and Tuina, Changchun University of Chinese Medicine, Northeast Asia Research Institute of Traditional Chinese Medicine, Changchun, China; d Department of Internal Medicine, Division of Brain Diseases, Affiliated Hospital of Changchun University of Chinese Medicine, Changchun, China.

**Keywords:** blood-activating herbs, edaravone, ischemic stroke, meta-analysis, protocol

## Abstract

Although the combination of blood-activating herbs and western drugs has shown advantages in the treatment of ischemic stroke, there is no consensus on the safety and efficacy. This study aimed to systematically evaluate the safety and efficacy of the combination of blood-activating herbs with edaravone (EDA) in the treatment of acute ischemic stroke (AIS).

**Methods:**

We will implement the search strategy in 8 English and Chinese databases: Cochrane Central Register of Controlled Trials, Web of Science, PubMed, China National Knowledge Infrastructure, China Science and Technology Journal Database, Wanfang Database, EMBASE and MEDLINE. The search included relevant clinical randomized controlled trials and quasi-randomized controlled trials that had been registered for publication by November 2022. Literature screening, data extraction and quality assessment will be performed by 2 authors. We will assess the risk of bias according to the Cochrane Handbook for Systematic Reviews of Interventions. The Grading of Recommendations Assessment, Development, and Evaluation (GRADE) method classification will be used to assess the quality of the literature. Meta-analysis was performed using RevMan V.5.4 and STATA 16 software.

**Results:**

This study will provide a comprehensive analysis of the current clinical evidence on the application of blood-activating herbs combined with EDA in the treatment of AIS.

**Conclusion:**

This study will clarify the safety and efficacy of the combination of blood-activating herbs with EDA in the treatment of AIS.

## 1. Introduction

Stroke is ranked as the second leading cause of death and the third leading cause of disability worldwide. With an annual mortality rate of approximately 5.5 million and long-term disability in up to 50% of stroke survivors, stroke is a major global public health problem that imposes a heavy burden on society and the economy.^[1,2]^ Epidemiological studies have shown that ischemic stroke accounts for more than 80% of all strokes in both developed and developing countries and is the predominant subtype of stroke.^[3]^ Currently, effective treatments for ischemic stroke include early restoration of cerebral perfusion and salvage of the ischemic semidark zone.^[4]^

Tissue-type fibrinogen activator (rt-PA, alteplase) is the only approved therapeutic agent for revascularization and improvement of neurological deficits. However, it is limited by the time window and the risks of hemorrhagic transformation and neurotoxicity, making it possible to benefit from it in only 2% to 5% of cases.^[5]^In addition to intravenous thrombolysis, treatment of acute ischemic stroke (AIS) includes control of blood glucose and blood pressure, prevention of increased intracranial pressure, and neuroprotective medications. Studies have noted that neuroprotective medications can play a beneficial role in patients who are not candidates for intravenous thrombolysis.^[6]^ The free radical scavenger edaravone (EDA) is one of the neuroprotective drugs, which is widely used in clinical practice in China, Japan, India and other countries. There is evidence that EDA significantly improves neurological outcomes in patients with AIS and has a safety profile that warrants promotion in the clinical management of AIS in Asian countries.^[7,8]^

Chinese herbal medicine is recommended by guidelines for secondary prevention of ischemic stroke, and its application in clinical practice to activate blood circulation and resolve blood stasis reduces the 2-year stroke recurrence rate in patients with ischemic stroke and does not increase the risk of bleeding in high-risk patients.^[9]^ The combination of blood-activating herbs and western medicine can effectively improve the quality of daily life of patients, and the combination of Chinese and Western medicine has advantages in the clinical treatment of ischemic stroke.^[10]^ Based on this, we conducted this systematic evaluation to assess the efficacy and safety of combining EDA with herbs that activate blood circulation and resolve blood stasis in the treatment of acute cerebral infarction, and to provide a basis for clinical decision making.

## 2. Methods

Our program will be conducted in strict compliance with the Preferred Reporting Items Guidelines for Systematic Reviews and Meta-Analysis Programs (PRISMA-P). The protocol is registered on PROSPERO under the registration number (CRD42022372004). As this is a literature-based study, an ethics statement is not required.

### 2.1. Inclusion criteria

#### 2.1.1. Types of studies.

Studies were included in randomized controlled trials (RCTs) and quasi-randomized controlled trials to evaluate the clinical efficacy and safety of EDA in combination with blood-activating herbs for the treatment of acute ischemic stroke. Studies were not subject to language restrictions and included all eligible trials. Non-compliant cohort studies, retrospective studies, reviews and animal cell trials will be excluded.

#### 2.1..2. Types of participants.

Inclusion criteria: Patients who met the diagnostic criteria for cerebrovascular disease set by the World Health Organization and were diagnosed with AIS by cranial CT or MRI; age and gender were not limited, and the onset of the disease was within 72 hours.

Exclusion criteria: patients who were not diagnosed with AIS; those with intracranial hemorrhage on transcranial CT or MRI; those who had an onset time of more than 72 hours; and those who did not have residual symptoms of neurological deficits onset.

#### 2.1.3. Types of interventions.

Patients in the intervention group were those who received conventional treatment and the combination of blood-stasis-activating herbs (herbal compound, Chinese patent medicine, injection) and EDA; those in the control group were those who received conventional treatment, or those who received conventional treatment in combination with blood-activating drugs, or those who received conventional treatment in combination with EDA.

#### 2.1.4. Types of outcomes.

Primary outcome indicators include the National Institutes of Health Stroke Scale; activities of daily living score (ADL); Barthel Index.

Secondary outcome indicators include blood rheology indicators, physical and chemical examination indicators, imaging indicators and the occurrence of adverse reactions.

### 2.2. Data sources and search methods

#### 2.2.1. Electronic searches.

We will implement the search strategy in 8 English and Chinese databases: Cochrane Central Register of Controlled Trials, Web of Science, PubMed, China National Knowledge Infrastructure (CNKI), China Science and Technology Journal Database, Wanfang Database, EMBASE and MEDLINE. The search was conducted for studies published up to November 2022.

#### 2.2.2. Search strategy.

The search strategy was guided by the Cochrane Handbook guidelines and included medical subject headings (MeSH) and variants. We will search for: acute ischemic stroke, acute cerebral infarction, stroke, cerebrovascular disease, Chinese medicine, herbal medicine, “Chinese herbal medicine, blood-activating herbs, EDA and all related subject terms. The detailed search strategy of Pubmed is shown in Table 1 and will be modified according to different databases (Table 1).

**Table 1:** PubMed search strategy.

Number	Search terms
#1	“Chinese medicine”[All Fields] OR “herbs”[All Fields] OR “traditional chinese medicine”[All Fields] OR “blood-activating herbs”[All Fields]OR “herbal medicine”[All Fields] OR “Chinese herbs”[All Fields] OR “Activating blood circulation herbs “[All Fields] OR “Activating blood circulation method”[All Fields]
#2	“Stroke”[All Fields] OR “ischemic stroke” [All Fields] OR “acute ischemic stroke”[All Fields] OR “cerebral ischemic stroke”[All Fields] OR “ischemic cerelral infarction” [All Fields] OR “cerebral infarction” [All Fields] OR “acute cerebral infarction” [All Fields] OR “ACI” [All Fields] OR “brain infarction” [All Fields] OR “Cerebrovascular disease” [All Fields] OR “Apoplexy” [All Fields] OR “Brain embolism” [All Fields] OR “Embolic stroke” [All Fields]
#3	“Edaravone”[All Fields] OR “frequency electromagnetic field ”[All Fields] OR “Edaravone Injection ”[All Fields]
#4	#1 AND #2 AND #3
#5	“randomized controlled trial” [Publication Type] OR “controlled clinical trial” [Publication Type] OR “Single-Blind Method” [Text Word] OR “Double-Blind Method” [Text Word] OR “random allocation” [Text Word] OR “allocation” [Text Word] OR “RCT” [Text Word] OR“Quasi- randomized controlled trial”[Text Word]
#6	#1 AND #4 AND #5

### 2.3. Data extraction and management

**Figure 1.**
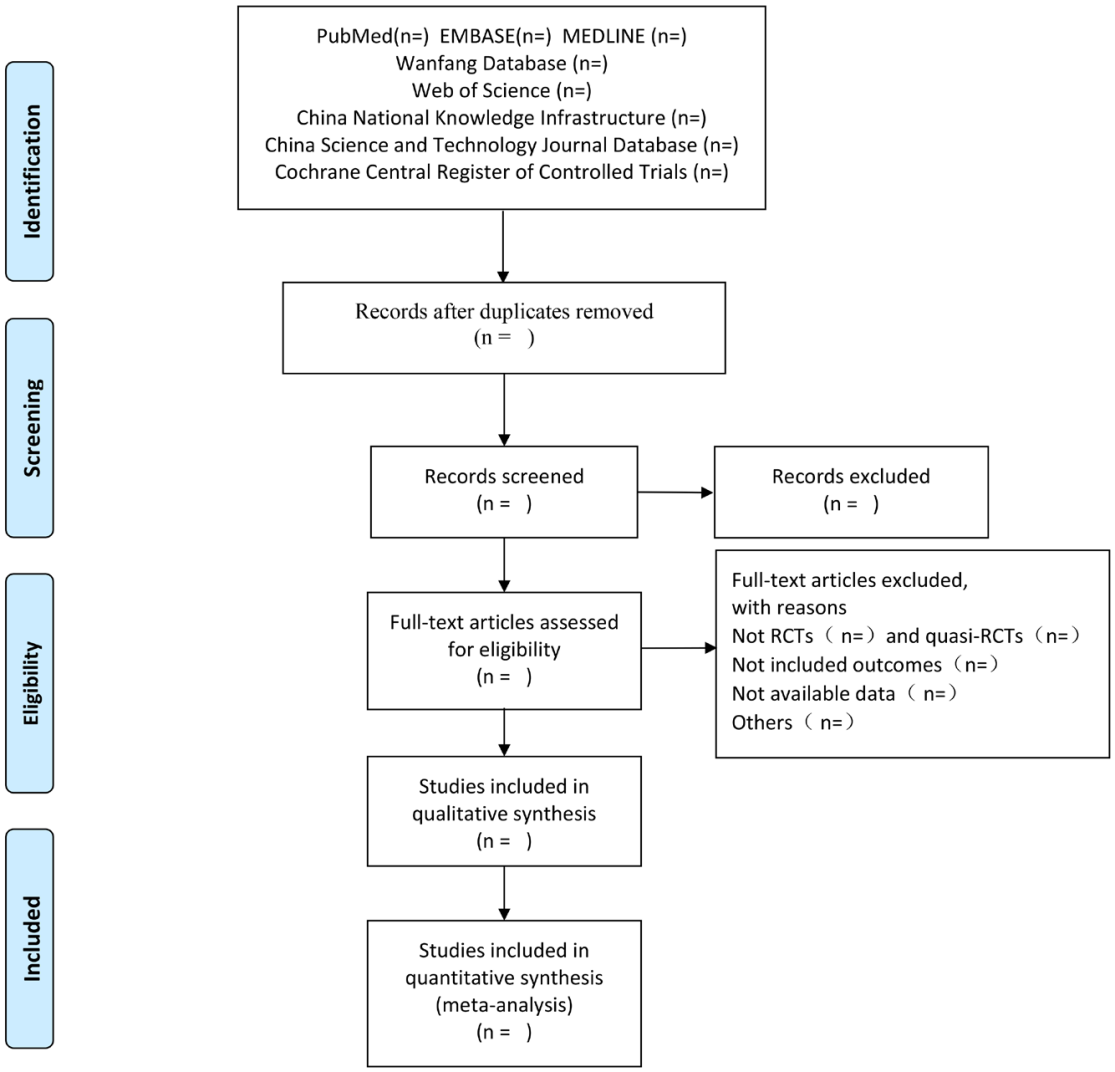
The screening process. RCT = randomized controlled trial.

Both authors will independently evaluate the titles, abstracts, and keywords of all citations found in the above search strategy to obtain the full text of all potentially appropriate articles. For missing data, we will contact the authors of the paper by email or phone to obtain them, and if data are not available in full, then the study will be excluded. Eligible literature was further screened based on the inclusion and exclusion criteria. For the screened literature, we also extracted the following information: general information (journal, time of publication, language), patient information (gender, age, time of onset), study design information (sample size, intervention method, intervention duration), and trial results. Disagreements that arise during the screening process will be resolved by consensus or intervention by a third author. The screening process is shown in Figure 1.

### 2.4. Risk of bias assessment

The two authors will use the Cochrane Handbook for Systematic Reviews of Interventions to assess the risk of bias and trial quality in the selected literature. Six main areas will be evaluated: random sequence generation and allocation concealment, blinding of participants and personnel, incomplete outcome data, outcome assessment, selection of reported outcomes and other biases (including pre-sample size estimates and early stopping of trials). RevMan V.5.4 will be used to plot and count the results of these issues. Each item will be assessed according to low risk of bias, high risk of bias, and unclear risk of bias. Disagreements arising from the assessment process will be resolved through discussion by a third author to reach consensus.

### 2.5. Measures of treatment effect

Statistical analyses were performed using RevMan V.5.4 software. Differences in means for continuous variables will be expressed as standard deviations, medians, or interquartile ranges. For dichotomous variables, we will report the overall mean proportion of the population (%). We will choose a 95% confidence interval to present the results for both continuous and dichotomous variables.

### 2.6. Heterogeneity assessment

Statistical heterogeneity will be assessed using the *I*^2^ statistic. 50% of the *I*^2^ value will be used as a cutoff for differentiation, if *I*^2^ < 50%, this indicates no significant heterogeneity between studies, this will be assessed using a fixed effects model. If *I*^2^ ≥ 50%, this indicates significant heterogeneity between studies, which will be assessed using a random effects model.

### 2.7. Assessment of reporting biases

We will assess publication bias or heterogeneity by Egger’s regression asymmetry test and symmetry of funnel plots.^[11,12]^ We will use STATA 16 software to perform the Egger test. Publication bias will not be explored if there are less than 10 published articles.

### 2.8. Analysis of subgroups or subsets

If heterogeneity is high, we plan to perform the following subgroup analyses to determine the source of heterogeneity: different dosage forms of blood-activating herbs, age (≥60 or <60), and different types of conventional medication use. In case of insufficient evidence, a qualitative rather than a quantitative synthesis will be performed.

### 2.9. Sensitivity analysis

We will perform sensitivity analysis by excluding low quality trials to check the robustness of the study results.

### 2.10. Grading of evidence quality

The quality of evidence regarding patient outcomes will be assessed by the Grading of Recommendations Assessment, Development, and Evaluation methodology (GREAD).^[13]^ The results of the quality of evidence assessment will be categorized into 4 grades: high, medium, low, and very low.

## 3. Discussion

With the increasing burden of cardiovascular disease, the incidence of AIS is also increasing year by year, becoming a frequent and common clinical disease. After AIS, cerebral artery embolism causes ischemia and hypoxia in the brain tissue of the affected area, triggering an ischemic cascade response. The normal transmembrane ion gradient and homeostasis maintained by neurons are disrupted, triggering a series of pathological processes such as excitotoxicity, oxidative stress, nitrative stress, and inflammation, resulting in neuronal cell necrosis and apoptosis. A series of symptoms such as neurological dysfunction is induced.^[14]^ Studies have shown that multiple pathways of injury in the ischemic cascade are simultaneous and interact with each other; therefore, combined therapy targeting several pathways of ischemic injury may have advantages over single pathway strategies.^[15]^

Free radicals are the main cause of ischemic cerebrovascular injury, and the massive production of free radicals after brain tissue injury promotes secondary brain tissue damage. EDA is a potent free radical scavenger. Studies have confirmed that EDA can effectively scavenge reactive oxygen species (ROS) in neutrophils, remove oxidative damage to neuronal cells, endothelial cells and brain cells by free radicals, and reduce tissue damage caused by acute cerebral infarction.^[16,17]^ EDA also has anti-inflammatory effects, reducing the damage to nerve cells by inflammatory factors, increasing cerebral blood flow, reducing neurological damage and improving prognosis.^[18]^ A retrospective clinical study showed that EDA improved the symptoms of neurological deficits in any ischemic stroke subtype and could exert neuroprotective effects when applied in the clinical treatment of ischemic stroke.^[19,20]^ Blood-activating herbs have unique advantages and good application prospects in the prevention and treatment of ischemic stroke. Several experimental studies have shown that blood-activating herbs and their active ingredients can promote neurogenesis and angiogenesis in rats with ischemic stroke and prevent the destruction of the blood-brain barrier after stroke.^[21,22]^ And can rescue tissue cells in the ischemic semidark zone through anti-inflammatory and anti-apoptotic mechanisms.^[23]^

The treatment of AIS with blood-activating herbs combined with EDA is widely used in the clinical treatment of cerebrovascular disease in China. Several RCTs have confirmed the effectiveness of the combination therapy, but there is a lack of systematic scientific evidence to support this conclusion. In this study, we will conduct a systematic review and meta-analysis of RCTs on the treatment of acute ischemic stroke with blood-activating and stasis-transforming herbs combined with EDA to verify its efficacy and safety and provide evidence-based medical evidence for the combined treatment of cerebrovascular disease with Chinese and Western medicine.

## Author contributions

**Data curation:** Zhuoyi Su, Hong Jin.

**Formal analysis:** Zhuoyi Su, Ziqiao Yu.

**Funding acquisition:** Dexi Zhao.

**Investigation:** Ziqi Yang.

**Methodology:** Weichen Sun.

**Validation:** Shuai Zhang.

**Writing – original draft:** Zhuoyi Su, Ziqiao Yu.

**Writing – review & editing:** Shuai Zhang, Dexi Zhao.
